# Occult Endobronchial Foreign Body Presenting as Persistent Lobar Collapse and Severe Pneumonia

**DOI:** 10.1002/rcr2.70466

**Published:** 2026-01-12

**Authors:** Venkatkiran Kanchustambham

**Affiliations:** ^1^ Pulmonary and Critical Care Medicine, Sanford Health and University of North Dakota School of Medicine and Health Sciences Fargo North Dakota USA

**Keywords:** airway obstruction, bronchoscopy, foreign body aspiration, lobar collapse, pneumonia

## Abstract

Foreign body aspiration is uncommon in adults and may present without a clear aspiration history. We describe an adult patient with persistent lobar collapse and severe pneumonia in whom bronchoscopy revealed an occult endobronchial foreign body causing airway obstruction.

## Graphical abstract

An adult patient with persistent lobar collapse and severe pneumonia was found to have an occult endobronchial foreign body on bronchoscopy. This case highlights the importance of considering foreign body aspiration in adults with non‐resolving pneumonia or unexplained lobar collapse.

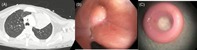

A 50‐year‐old man with intellectual disability, autism spectrum disorder, epilepsy, hypothyroidism and diabetes mellitus presented with altered mental status and severe pneumonia. Chest computed tomography (CT) demonstrated complete left upper lobe collapse with an abrupt cutoff of the left upper lobe bronchus, raising concern for an obstructing endobronchial lesion (Figure 1A). Despite appropriate antibiotic therapy, the patient showed minimal clinical improvement.

**FIGURE 1:**
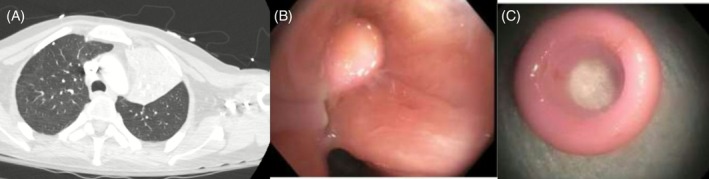
Composite image demonstrating an occult endobronchial foreign body. (A) Chest computed tomography showing complete left upper lobe collapse with abrupt airway cutoff. (B–C) Bronchoscopic views demonstrating an impacted foreign body obstructing the left upper lobe bronchus with surrounding mucosal inflammation.

Flexible bronchoscopy revealed an impacted endobronchial foreign body completely obstructing the left upper lobe bronchus, with surrounding mucosal inflammation and friability (Figure 1B,C). The foreign material was successfully removed bronchoscopically, resulting in immediate restoration of airway patency and subsequent clinical and radiographic improvement. No active bleeding was identified during bronchoscopy.

Foreign body aspiration in adults is uncommon and frequently occurs without a witnessed aspiration event, particularly in patients with neurologic or cognitive impairment [1]. Persistent lobar collapse or non‐resolving pneumonia should prompt evaluation for endobronchial obstruction even in the absence of choking history. Early bronchoscopy is both diagnostic and therapeutic and can prevent recurrent infection and long‐term pulmonary complications [2].

## Author Contributions

Venkatkiran Kanchustambham was responsible for patient care, image selection, literature review and manuscript preparation.

## Funding

The author has nothing to report.

## Consent

The author declares that written informed consent was obtained for the publication of this manuscript and accompanying images using a consent form that complies with the Journal's requirements as outlined in the author guidelines.

## Conflicts of Interest

The author declares no conflicts of interest.

## Data Availability

The data that support the findings of this study are openly available in google scholar at https://scholar.google.com/citations?view_op=list_works&hl=en&hl=en&user=JGwxTD0AAAAJ&sortby=pubdate.
